# Dehulled Adlay Consumption Modulates Blood Pressure in Spontaneously Hypertensive Rats and Overweight and Obese Young Adults

**DOI:** 10.3390/nu13072305

**Published:** 2021-07-04

**Authors:** Wan-Ju Yeh, Jung Ko, Wei-Yi Cheng, Hsin-Yi Yang

**Affiliations:** 1 Graduate Program of Nutrition Science, National Taiwan Normal University, Taipei 106308, Taiwan; wandayeh@ntnu.edu.tw; 2 Department of Applied Biological Chemistry, Graduate School of Agricultural and Life Sciences, The University of Tokyo, Tokyo 113-8657, Japan; 532732002@hotmail.com; 3 Department of Nutrition, I-Shou University, Kaohsiung City 824005, Taiwan; wicheng@isu.edu.tw; 4 Department of Nutritional Science, Fu Jen Catholic University, No.510, Zhongzheng Rd., Xinzhuang Dist., New Taipei City 242062, Taiwan

**Keywords:** adlay, hypertension, blood pressure, ACE, ET-1

## Abstract

High blood pressure is a crucial risk factor for many cardiovascular diseases, and a diet rich in whole-grain foods may modulate blood pressure. This study investigated the effects of dehulled adlay consumption on blood pressure in vivo. We initially fed spontaneous hypertensive rats diets without (SHR group) or with 12 or 24% dehulled adlay (SHR + LA and SHR + HA groups), and discovered that it could limit blood pressure increases over a 12-week experimental period. Although we found no significant changes in plasma, heart, and kidney angiotensin-converting enzyme activities, both adlay-consuming groups had lower endothelin-1 and creatinine concentrations than the SHR group; the SHR + HA group also had lower aspartate aminotransferase and uric acid levels than the SHR group did. We later recruited 23 participants with overweight and obesity, and they consumed 60 g of dehulled adlay daily for a six-week experimental period. At the end of the study, we observed a significant decrease in the group’s systolic blood pressure (SBP), and the change in SBP was even more evident in participants with high baseline SBP. In conclusion, our results suggested that daily intake of dehulled adlay had beneficial effects in blood-pressure management. Future studies may further clarify the possible underlying mechanisms for the consuming of dehulled adlay as a beneficial dietary approach for people at risk of hypertension.

## 1. Introduction

Hypertension is associated with mortality and morbidity related to various cardiovascular diseases [1]. Long-term high blood pressure may lead to cardiorenal remodeling and increased the risk of tissue injuries [2]. In a 2014 evidence-based guideline on the management of adult high blood pressure, adults with systolic blood pressure (SBP) and diastolic blood pressure (DBP) greater than 140 and 90 mmHg, respectively, were determined to have hypertension [3]. In addition, according to the American College of Cardiology/American Heart Association (ACC/AHA) 2017 Guideline for the Prevention, Detection, Evaluation, and Management of High Blood Pressure in Adults, adults with SBP and DBP above 130 and 80 mmHg, respectively, were defined as having hypertension, and individuals with SBP of 120–139 mmHg and DBP less than 80 mmHg were defined as having elevated blood pressure [4]. These guidelines served to increase awareness of hypertension; encourage proper antihypertensive medication usage, such as medications blocking the renin-angiotensin system; and lifestyle modification, such as adherence to the Dietary Approaches to Stop Hypertension (DASH) recommendations [5].

According to a 2019 meta-analysis survey, dietary consumption of whole grains rather than refined grains may aid in the prevention of non-communicable diseases [6]. The prospective study demonstrated that greater consumption of whole grains decreased the risk of hypertension in the Japanese population [7]. The blood-pressure-lowering DASH diet also recommends whole-grain foods because they contain fiber, minerals, vitamins, and other bioactive chemical components with health benefits [8,9]. Adlay (*Coix lachryma-jobi* L. var. *ma-yuen* Stapf) is a popular grain in Asian cuisine, and has been used as a traditional Chinese medicine for its antioxidative and anti-inflammatory potential [10]. Recent studies have also reported that adlay bran, which is rich in phenolic compounds, had beneficial effects on lipid metabolism and inflammatory responses in vivo [11,12]. Daily consumption of 60 g of adlay was also found to improve plasma lipid profiles in hyperlipidemic male patients [13]. However, evidence of adlay’s effectiveness in blood-pressure modulation remains limited. Only one study reported that enzymatic hydrolyzed peptides derived from adlay seed exhibited the potential to inhibit angiotensin-converting enzyme (ACE) activity and to reduce blood pressure in rats [14]. Therefore, we aimed to investigate the effects of a diet rich in dehulled adlay instead of other refined cereals on blood-pressure regulation in both spontaneously hypertensive animals and in overweight and obese adults.

## 2. Materials and Methods

### 2.1. Animal Study

We purchased dehulled adlay powder (Taichung No. 3) from the Nantou County Tsao-Tun Production Association (Nantou County, Taiwan). Eight-week-old Wistar Kyoto (WKY) rats and spontaneously hypertensive rats (SHRs) were purchased from the National Laboratory Animal Breeding and Research Center. Rats were housed in the Experimental Animal Center as per guidelines reviewed by the Institutional Animal Care and Use Committee of I-Shou University (Approval ID: AUP-105-43-01). Rats were maintained in an environment with a constant temperature (23 ± 2 °C) and humidity (55 ± 10%) and were exposed to a 12 h light–dark cycle in accordance with the Animal Protection Act and the regulations of the Animal Care and Use Committee of the Council of Agriculture, Executive Yuan. Rats were fed a standard rat chow diet for 1 week for acclimatization. Then, SHRs were randomly assigned to three groups: an SHR group fed a standard AIN-93M rodent diet (*n* = 10), an SHR + LA group fed AIN-93M with a low dose of 12% adlay powder (*w*/*w*), and an SHR + HA group fed AIN-93M with a high dose of 24% adlay powder (*w*/*w*), WKY rats were used as the normotensive control and also received a standard AIN-93M rodent diet (*n* = 10). We used adlay powder substituted for part of the components from the standard AIN-93M diet to ensure equal nutrient composition among the diets, as shown in Table 1. During the 12-week experimental period, food and water were provided ad libitum. Rats’ food intake was recorded daily and their body weights recorded weekly. At the end of the study, we collected the previous 24 h of urine of rats using metabolic cages, and thereafter sacrificed the rats to obtain blood, heart, and kidney samples for analysis.

#### 2.1.1. Measurement of Blood Pressure

SBP and DBP were measured using a noninvasive tail-cuff system (MK-2000ST, Muromachi Kikai, Tokyo, Japan) every 4 weeks. Rats were placed in restrainers, and we recorded at least five readings to calculate the mean of blood pressure over the course of the measurement.

#### 2.1.2. Blood Analysis

Blood samples were collected and centrifuged at 1500× *g* and 4 °C for 15 min for plasma separation. Plasma samples were collected to analyze the aspartate aminotransferase (AST), alanine aminotransferase (ALT), creatinine (Cr), uric acid, and phosphorus concentrations by using a Roche Modular P800 Autoanalyzer (Diagnostics Roche, Basel, Switzerland). ACE activity was analyzed according to the method previously described by Vermeirssen et al. [15]. C-reactive protein (CRP; Invitrogen, CA, USA), plasminogen activator inhibitor-1 (PAI-1; HYPHEN BioMed, Neuville-sur-Oise, France), and endothelin-1 (ET-1; Enzo Life Sciences, New York, USA) were analyzed using commercial kits as per the manufacturer’s instructions.

#### 2.1.3. Urine Analysis

The 24 h of urine samples were collected using metabolic cages. Urinary protein excretion, urine urea nitrogen (UUN), and Cr levels were analyzed using a Roche Modular P800 Autoanalyzer (Diagnostics Roche, Basel, Switzerland). All urine values were corrected in accordance with the Cr level.

#### 2.1.4. Heart and Kidney Analysis

Heart and kidney samples were homogenized in a buffer solution (50 mM Tris-HCl, 150 mM NaCl, 0.1% SDS, 1% NP-40, pH 7.5), and suspensions were centrifuged at 1000× *g* at 4 °C for 10 min. ACE activity levels of the hearts and kidneys were determined using the method described by Yang et al. [16].

### 2.2. Human Study

**Figure 1:**
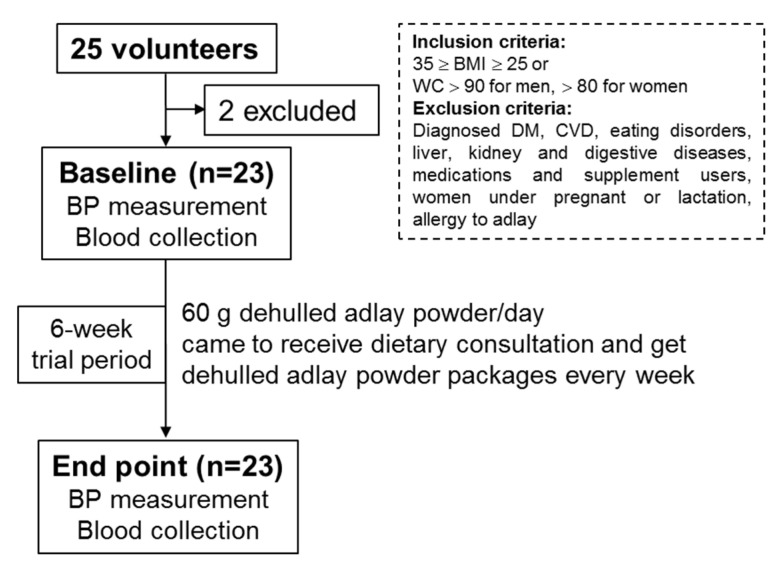
Flowchart illustrating the number of volunteers who were screened, included, and completed the study.

Participants were recruited through announcement posters at the I-Shou University (Kaohsiung, Taiwan). People with diagnoses of diabetes, cardiovascular diseases, eating disorders, or liver, kidney, or other digestive diseases; medications and supplement users; pregnant or lactating individuals; and people with an adlay allergy were excluded. We recruited 25 volunteers, and among them, 23 participants with a body mass index (BMI, kg/m^2^) within the range of 25 to 35 (inclusive) or waist circumference within an indicated sex-specific range (>90 cm for men and >80 cm for women) were included in the experiment as shown in Figure 1. We explained the purpose and design and of the study, as well as the risks involved to all participants, and obtained their written informed consent.

#### 2.2.1. Experimental Design

We implemented this study following the protocol approved by the Institutional Review Board (IRB) of E-Da Hospital according to the guidelines in the Declaration of Helsinki (IRB No.: EMRP41104N). During the 6-week experimental period, 23 participants were asked to consume two packages (30 g/package) of dehulled adlay powder per day to replace three corresponding portions of cereal, according to the Food Exchange List of Taiwan and the dosage consumed daily in a previous study [13]. Those participants came to our center weekly to receive dietary consultation, bring back the used container, and obtain their adlay powder packages for the coming week. During the experimental period, the consumption of other supplements and foods not specified in the study was prohibited, and all participants were asked to maintain their normal dietary habits and physical activities.

#### 2.2.2. Blood Pressure Measurement

Participants visited our research center at 07:30 a.m. at the baseline and the end of the study after a fasting period of at least 8 h. Blood pressure was measured using an automatic blood-pressure monitor on their right arm (Microlife, Taipei, Taiwan) after participants sat in a chair for at least 10 min. SBP and DBP were calculated as the average of three separate measurements.

#### 2.2.3. Blood Analysis

At the baseline and end of the study, we collected plasma samples after blood-pressure measurement to measure ET-1 concentrations as described before.

### 2.3. Statistical Analysis

All data are presented as mean ± standard deviation (SD). Data from the animal study, and on changes in blood pressure among the three subgroups according to the guideline [4] (<120, 120–130, >130 mmHg) in the human study, were analyzed using one-way analysis of variance (ANOVA) with a post hoc Tukey test in SAS version 9.3. The differences in body weight and in blood pressure of animals at different time points and among groups were analyzed using repeated-measures ANOVA and Duncan’s multiple-range test. The blood pressure and plasma ET-1 levels of participants at baseline and the end of the study were evaluated using a paired *t*-test. A *p*-value of <0.05 indicated a statistically significant difference.

## 3. Results

### 3.1. Effects of Dehulled Adlay Intake on Body Weight in SHRs

**Figure 2:**
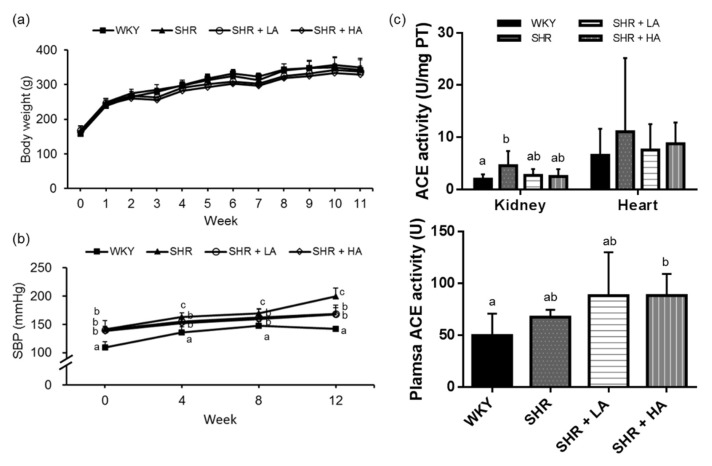
Food intake (**a**), SBP (**b**), and plasma and tissue ACE activities (**c**) of rats in different groups. Values are presented as mean ± SD (*n* = 10). ^abc^ Different superscript letters indicate a significant difference (*p* < 0.05). SBP, systolic blood pressure; ACE, angiotensin-converting enzyme. WKY, Wistar Kyoto) rats fed an AIN-93M diet; SHR, spontaneously hypertensive rats fed an AIN-93M diet; SHR + LA, SHRs fed an AIN-93M diet containing 12% dehulled adlay powder; SHR + HA, SHRs fed an AIN-93M diet containing 24% dehulled adlay powder.

The results of the animal experiment demonstrated that using a diet containing dehulled adlay did not affect food and energy intake among groups. In addition, we also discovered no significant differences in body weight among the groups over the experiment period (Figure 2a).

### 3.2. Effects of Dehulled Adlay Intake on Blood Pressure and ACE Activity in SHRs

The SHR, SHR + LA, and SHR + HA groups all had higher SBP levels than the WKY group throughout the 12-week experimental period. Both the SHR + LA and SHR + HA groups had lower SBP than the SHR group after the fourth week, and this difference was maintained until the end of the study (Figure 2b). Although the SHR group had higher renal ACE activity than the WKY group (WKY vs. SHR, *p* = 0.0004), we found no significant difference in plasma, heart, and kidney ACE activity among the SHR, SHR + LA, and SHR + HA groups (Figure 2c). The results indicated that daily dehulled adlay intake in replacement of part of the diet composition could prevent blood-pressure increases among SHRs, but these effects may not be explained by the inhibition of ACE activity alone.

### 3.3. Effects of Dehulled Adlay Intake on AST, ALT, and Renal Functions in SHRs

**Figure 3:**
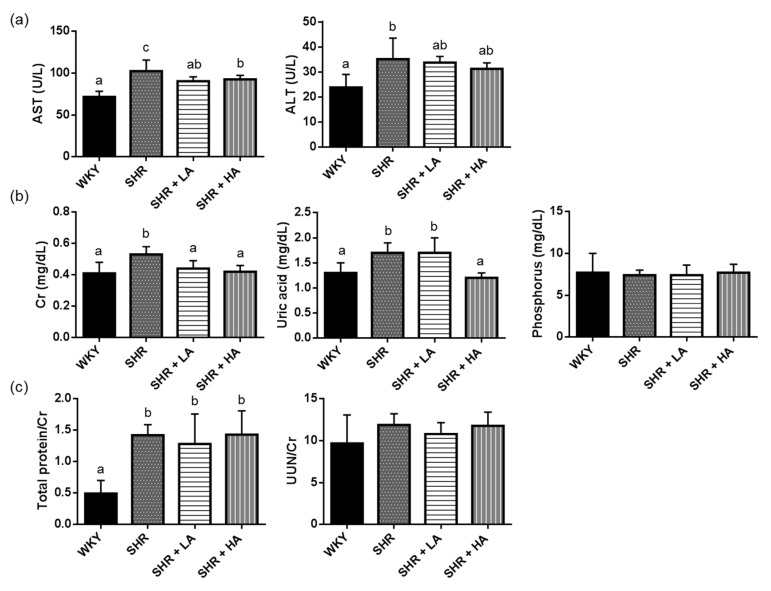
Plasma hepatic (**a**), renal function parameters (**b**), and urine analysis (**c**) of rats in different groups. Values are presented as mean ± SD (*n* = 10). ^abc^ Different superscript letters indicate a significant difference (*p* < 0.05). AST, aspartate aminotransferase; ALT, alanine aminotransferase; UUN, urine urea nitrogen. WKYWistar Kyoto rats fed an AIN-93M diet; SHR, spontaneously hypertensive rats fed an AIN-93M diet; SHR + LA, SHRs fed an AIN-93M diet containing 12% dehulled adlay powder; SHR + HA, SHRs fed an AIN-93M diet containing 24% dehulled adlay powder.

At the end of the study, we found that the SHR group had higher plasma AST (WKY vs. SHR, *p* < 0.0001; SHR + LA vs. SHR, *p* = 0.0011; SHR + HA vs. SHR, *p* = 0.0076) and ALT (WKY vs. SHR, *p* < 0.0001) activities than the WKY group did, and that both the SHR + LA and SHR + HA groups had lower AST activities than the SHR group did (Figure 3a). In addition, plasma Cr concentrations (WKY vs. SHR, *p* = 0.0289) and the total protein-to-Cr ratios in the rat urine were significantly higher in the SHR groups than in WKY (WKY vs. SHR, *p* = 0.0042). Both dehulled adlay groups had lower plasma Cr concentrations than the SHR group did (SHR + LA vs. SHR, *p* = 0.0020; SHR + HA vs. SHR, *p* < 0.0001), but no significant differences were observed in total protein/Cr, UUN/Cr, and plasma phosphorus concentrations in urine among the three SHR groups. Additionally, plasma uric acid levels were significantly higher in the SHR group and lower in the HA group (WKY vs. SHR, *p* = 0.0022; SHR + HA vs. SHR, *p* = 0.0002) (Figure 3b,c). The results also indicated that elevation of blood pressure may increase the risk of renal tissue injuries, and that dehulled adlay consumption may ameliorate these risks.

### 3.4. Effects of Dehulled Adlay Intake on Indicators of Endothelial Function in SHRs

**Figure 4:**
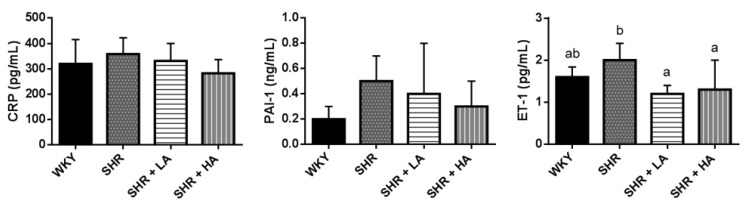
Plasma CRP, PAI-1, and ET-1 levels of rats in different groups. Values are presented as the mean ± SD (*n* = 10). ^ab^ Different superscript letters indicate a significant difference (*p* < 0.05). CRP, C-reactive protein; PAI-1, plasminogen activator inhibitor-1; ET-1, endothelin-1. WKYWistar Kyoto rats fed an AIN-93M diet; SHR, spontaneously hypertensive rats fed an AIN-93M diet; SHR + LA, SHRs fed an AIN-93M diet containing 12% dehulled adlay powder; SHR + HA, SHRs fed an AIN-93M diet containing 24% dehulled adlay powder.

Over the course of the experiment, plasma CRP and PAI-1 levels tended to decrease in both the SHR + LA and SHR + HA groups when compared with the SHR group. Both dehulled-adlay-consuming groups also had lower plasma ET-1 levels than did the SHR group (SHR + LA vs. SHR, *p* = 0.0164; SHR + HA vs. SHE, *p* = 0.0345), and no significant difference in ET-1 level was observed compared with the WKY group (Figure 4). The results showed that adlay may retard the elevation of blood pressure through improving endothelial function.

### 3.5. Effects of Daily Dehulled Adlay Intake on Blood Pressure and Endothelial Function in Participants

**Figure 5:**
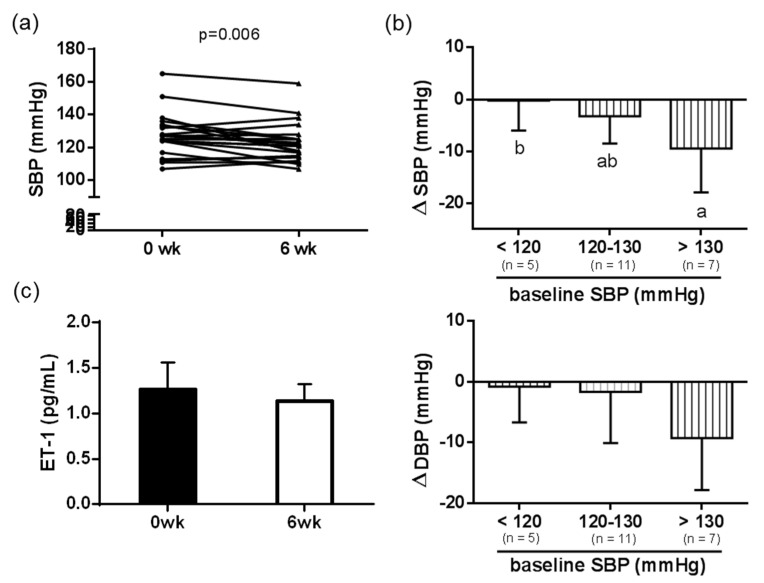
Baseline and end SBP (**a**) and ET-1 (**b**) levels, and changes in SBP and DBP (**c**) of participants subgrouping by baseline SBP after the 6-week dehulled adlay intervention. Values are presented as mean ± SD (*n* = 23). ^ab^ Different superscript letters indicate a significant difference (*p* < 0.05). SBP, systolic blood pressure; DBP, diastolic blood pressure; ET-1, endothelin-1.

Furthermore, we performed an interventional human study to observe the effects of a dehulled adlay-rich dietary pattern on blood-pressure regulation in participants. During the experimental period, participants were asked to maintain their normal dietary and physical activity, but to replace 60 g of refined grain products with dehulled adlay powder either in their beverage or meal under the guidance of a dietitian; subjects brought back the used and empty containers to our center every week, and no subjective adverse effects were reported. We learned that the SBP of our participants decreased over the 6-week experimental period (6-week vs. 0-week, *p* = 0.006) (Figure 5a). Additionally, we categorized participants into subgroups according to their baseline SBP (<120 mmHg, *n* = 5; 120–130 mmHg, *n* = 11; >130 mmHg, *n* = 7), and discovered that the effects of dehulled adlay consumption on blood-pressure change were more obvious in the participants with higher baseline SBP (∆SBP: >130 vs. <120, *p* = 0.0243) (Figure 5b). We also noted a trend of decreasing plasma ET-1 levels in participants (*p* = 0.07) (Figure 5c). These results indicated a beneficial effect of dehulled adlay on blood-pressure modulation, and that these effects may be related to baseline SBP.

## 4. Discussion

In this study, we found that dehulled adlay consumption retarded the elevation of blood pressure in SHRs, and may be beneficial in blood pressure modulation in overweight and obese adults. Dietary intake of whole-grain foods may lower cardiovascular risks. A randomized controlled trial concluded that daily consumption of whole grains (50 g/1000 kcal) resulted in greater improvements in blood pressure than a refined grain diet did in adults with overweight and obesity [17]. Results from the Furukawa Nutrition and Health Study also indicated that higher intake of whole-grain foods may reduce hypertension risk [7]. Adlay is a common grain in Asian diets, and is used in traditional Chinese medicines to treat cardiovascular diseases. Studies have also reported that adlay has various beneficial effects. For example, adlay bran was discovered to have anti-inflammatory [18] and anti-tumor effects [19], and dehulled adlay also had a gastroprotective effect in vitro [20]. Although one previous study reported that adlay-derived peptides may have antihypertensive effects [14], studies focused on adlay’s influence on blood-pressure management remains scarce. To our knowledge, this is the first study to explore the potential effects of daily consumption of dehulled adlay in reducing blood pressure in hypertensive rats and in overweight and obese participants.

Increased daily whole-grain consumption has positive effects on blood-pressure control. The 2020 International Society of Hypertension’s Global Hypertension Practice Guidelines also include a suggestion to eat a healthy diet rich in whole grains to treat hypertension [21]. Dehulled adlay is one of the ingredients recommended to replace polished rice in some Asian diets. According to the Nutrient Composition Database of the Food and Drug Administration of the Ministry of Health and Welfare, every 100 g of adlay seed contains 199 mg of magnesium, which is approximately 10 times of the level in white rice. Magnesium has been shown to regulate blood pressure through directly stimulating prostacyclin and nitric oxide production [22]. These blood-pressure-reducing effects may be caused by endothelium-dependent and endothelium-independent vasodilation [23,24]. Furthermore, magnesium may also prevent vascular injury due to its antioxidant and anti-inflammatory effects [25]. In the present study, we discovered that in hypertensive rats, partial dietary replacement with dehulled adlay could limit the progression of hypertension without affecting food intake or body weight in hypertensive rats. We also found that daily intake of 60 g dehulled adlay could lower blood pressure in human participants with high baseline SBP. These results suggested that dehulled adlay has potential use for the treatment or prevention of hypertension.

Blood pressure is regulated by numerous mechanisms in vivo, and the renin-angiotensin system is one of such major regulatory mechanism. Increased ACE activity reveals the formation of angiotensin II, which leads to vessel constriction and elevated blood pressure. In 2017, Li et al. [14] observed potent anti-hypertensive peptides in Coix glutelin. However, we found no significant effect of dehulled adlay on plasma, kidney, and heart ACE activities in SHRs; these rats did, however, have significantly lower SBP at the end of the 12-week experimental period. Therefore, the lowered blood pressure associated with consuming dehulled adlay cannot be explained by its ACE inhibitory activity. Conversely, we found that both dehulled adlay intervention groups had significant lower plasma ET-1 and Cr concentrations than the non-treated SHR group did. Studies have indicated that ET-1 secretion raises blood pressure and accelerates the progression of nephropathy by stimulating vasoconstriction and the retention of water and sodium [26]. Therefore, our results indicated that dehulled adlay may not only retard the elevation of blood pressure in hypertensive rats, but also reduce the risk of kidney injury. In addition, we discovered, through human trials, that replacing part of daily staple food intake with dehulled adlay intake for up to six weeks produced positive effects of lower blood pressure. However, no significant change in ET-1 was found six 6 weeks. On the basis of these results, future studies should extend the experimental period or increase the sample size to further clarify the mechanisms underlying such outcomes.

Recent studies have found that uric acid is strongly linked to high blood pressure. A cross-sectional study reported that each 1 mg/dL increase in plasma uric acid increases the risk of hypertension by 20% [27]. Uric acid may directly cause endothelial dysfunction. When uric acid crystals are deposited in blood vessels, vascular inflammation and endothelial damage arise [28]. Moreover, uric acid could also affect vascular function through crystalline-independent pathways. Otani et al. [29] revealed that uric acid has the potential to reduce the phosphorylation of endothelial nitric oxide synthase and to damage endothelial function. Furthermore, hyperuricemia leads to increased ET-1 expression and renal injury [30]. In a hyperuricemic rat model, dehulled adlay extract effectively decreased serum uric acid levels by inhibiting xanthine oxidase [31]. In this study, we observed that rats fed high-dose dehulled adlay exhibited lower plasma uric acid levels than the SHR group, but the level was not significantly different from those of the WKY group. In addition, we ascertained that levels of the inflammatory-response indicators CRP and PAI-1 tended to decline as a result of consumption of an adlay-rich diet. These results indicated that the blood-pressure reduction associated with dehulled adlay may be related to moderating effects on uric acid and ET-1 levels.

This is the first study to apply dehulled adlay intake in the daily diet of participants with a high risk of hypertension, and we observed that replacing 60 g of staple food in daily diet with dehulled adlay helped moderate high blood pressure. In the animal study, we also discovered that dehulled adlay intake curbed blood-pressure elevation and lessened uric acid and ET-1 levels. However, some limitations were present in our study and may be rectified by further studies. First, we used a non-invasive tail-cuff method with a four-week interval to investigate the change of blood pressure in this study. A telemetry system may be more ideal, and could be used to record more hemodynamic information in future long-lasting experiments. Second, the number of human participants in this study was limited and lacked a control group. The single-arm study design followed by a pre-post evaluation can only offer preliminary information, and may not completely exclude the placebo effects of the intervention [32]. Although there currently are not enough previous references available about the effects of adlay on blood pressure, the results of this study can be used as a basis for further research. Future studies may increase the number of participants, extend the duration of the intervention period, use a control group consuming refined cereals, and measure dietary intake throughout the intervention to better observe more influential results and to clarify the related underlying mechanisms. Third, we used participants with high risks of cardiovascular diseases, such as overweight and obesity, in this study, and discovered that the effects of dehulled adlay intake on blood-pressure reduction was more evident in participants with high basal blood pressure. Therefore, future studies also could focus on patients diagnosed as having hypertension to further explore whether combining drug treatment with daily dehulled adlay consumption would have synergistic effects. The pathways related to uric acid and ET-1 also could be emphasized in future investigations.

## 5. Conclusions

In conclusion, our results suggested that daily intake of 60 g dehulled adlay had beneficial effects on blood-pressure management. Future studies could further clarify the possible underlying mechanisms for the consuming of dehulled adlay as a beneficial dietary approach for people at risk of hypertension.

## Figures and Tables

**Table 1:** Dietary compositions (g/kg) of groups in the murine trial.

	WKY	SHR	SHR + LA	SHR + HA
Casein	140	140	118.2	96.3
Dextrin	155	155	155	155
Corn starch	465.7	465.7	389.4	313.1
Sucrose	100	100	100	100
Soy oil	40	40	38.4	36.9
Cellulose	50	50	29.7	9.4
L-cystine	1.8	1.8	1.8	1.8
AIN-93M mineral mix	35	35	35	35
AIN-93M vitamin mix	10	10	10	10
Choline bitartrate	2.5	2.5	2.5	2.5
Dehulled adlay	0	0	120	240

Corn starch, dextrin, casein, soy oil, cellulose (non-nutritive bulk), AIN-93M vitamin and mineral mixture were obtained from ICN Biochemicals (Aurora, OH, USA). Choline bitartrate and cystine were obtained from Sigma (St. Louis, MO, USA). Dehulled adlay (Taichung No. 3) powder was purchased from the Nantou County Tsao-Tun Production Association (Nantou County, Taiwan). SHR + LA, low-dose (12%, *w*/*w*) dehulled adlay powder in diet; SHR + HA, high-dose (24%, *w*/*w*) dehulled adlay powder in diet.
